# Achieving oral and planetary health; how sports dental medicine can pioneer and realize transformation in oral healthcare

**DOI:** 10.3389/froh.2025.1664261

**Published:** 2025-10-29

**Authors:** Stavros Avgerinos, Julian Fisher, Ivo Krejci, Aafreen Saiyed, Avijit Banerjee

**Affiliations:** 1 German Society for Sports Dentistry, Swiss Biological Medicine Center, SBMC AG, Kreuzlingen, Switzerland; 2 Oral and Planetary Health Policies, Center for Integrative Global Oral Health, University of Pennsylvania l School of Dental Medicine, Philadelphia, PA, United States; 3 Faculty of Medicine, University Dental Clinics, University of Geneva, Geneva, Switzerland; 4 Friede Springer Cardiovascular Centre, Charité University Berlin, Berlin, Germany; 5 Centre of Oral Clinical Translational Sciences, Faculty of Dentistry, Oral & Craniofacial Sciences, King’s College London, London, United Kingdom

**Keywords:** sports dentistry, sustainability, minimum intervention oral healthcare, artificial intelligence, planetary health, health promotion, transdisciplinary care, oral-systemic

## Abstract

The present manuscript is consistent with the objectives and scope of Frontiers in Oral Medicine, particularly the Oral Health Promotion section. The text presents a forward-looking perspective on how the potential of sports dental medicine can act as a catalyst for promoting sustainable and AI-enhanced oral healthcare. The article discusses a conceptual shift toward preventive, interprofessional and transdisciplinary collaboration, technology-integrated models of care, including key themes such as health promotion, system-level innovation and planetary health.

## 1 Introduction; current advances and future directions

Dentistry, like many other sectors, is facing a triple planetary crisis of climate change, nature and biodiversity loss, and pollution and waste (1). This requires a more comprehensive approach that positions dentistry within key decision-making fora and planning processes from local to global levels, while identifying positive tipping points that can advance sustainable oral health, where opportunities for beneficial changes become self-sustaining.

Sports medicine, also called sport and exercise medicine (SEM), is a recognised discipline concerned with physical fitness and prevention and treatment and of sports and exercise related injuries. Global disruptions like climate change, biodiversity loss, digital transformation, and sustainability integration are reshaping health systems and societal stability and are key drivers of transformation in the health sector, including sports medicine and dentistry. Sports medicine has evolved into a transdisciplinary field that considers medical advances, changing values and preferences, environmental and social determinants of health and planetary health including the impact of climate change. Sports medicine contributes to healthier athletes and better performance, where sports dentistry is recognised as an integral component.

Although considered medically healthy, elite athletes are a highly vulnerable population for oral diseases, presenting with dental caries (tooth decay; 49.1%) (ICDAS code ≥3), erosive dental lesions (41.4%), gingival inflammation (77.0%), periodontitis (gum disease; 21.6%) and bruxism (tooth grinding; 100%) (2). These conditions affect essential functions such as eating (34.6%), relaxation (15.1%) and smiling (17.2%). The reasons for these findings may be attributed to different factors including oxidative stress, sports diet and oral hygiene (3). The risks factors underlying these conditions are not unique to oral health, but are common to health in general.

New and emerging challenges such as climate change will amplify risks to the oral and general health of athletes particularly those who train outdoors and compete in outdoor venues. Climate change is associated with increases in health-related risks for athletes in particular: heat stress, UV exposure, exposure to allergens, exposure to air pollutants, spread of vectors and natural reservoirs as well as extreme weather events (4). These stressors will impact oral and lung function, musculoskeletal function and heighten physiological responses placing additional strain on organs such as the heart and kidneys. Consequently, the sports dentistry profession must reevaluate oral health assessment criteria and protocols to consider these challenges as they relate to oral and systemic health. This applies equally to athletes and the general population. In this way, adopting new ways of thinking and working towards sustainable oral health for athletes can pioneer and realise broader oral healthcare transformation.

## 2 From sports dentistry to sports dental medicine

Sports dentistry traditionally addressed prevention and treatments of the pathologies and injuries of the oral cavity and the stomatognathic system related to sports practise. However, the prevailing mindset is that oral health is synonymous with dentistry and that poor oral health has little impact on personal and societal health and wellbeing (5). New definitions of oral health provide an opportunity to change mindsets and promote innovation in sports dentistry.

The World Health Organization's definition of oral health includes breathing, which frames oral health within lung health and reinforces the importance of oral health in enabling individuals to perform essential functions. The FDI World Dental Federation model of oral health describes three interconnected domains: i. disease and condition status, ii. physiological status and iii. psychosocial function (6). FDI includes the craniofacial complex (head, face and oral cavity) that connects to the musculoskeletal system, an essential component of human health and performance. These shifts in definitions can catalyse a transformation from sports dentistry based on a biomedical restorative-orientated model of care to sports dental medicine that is grounded in the concept of sustainable oral health within a framework for oral and planetary health.

The paper aims to describe how sports dental medicine can have a pioneering role in the evolution of the profession and the broader transformations needed in the oral healthcare system.

The authors propose that the rapidly growing possibilities of Artificial Intelligence (AI), digital developments, Minimum Intervention Oral Care (MIOC), biomimetic technologies can provide a blueprint to guide and inform transformation in sports dentistry and clinical dental practice.

### 2.1 Sustainable oral health vs. a reactive model

Traditionally sports dentistry has been viewed through the lens of oral diseases and restorative dentistry and founded on a reactive model, i.e., waiting for disease to manifest before implementing resource-intensive interventions. This is exemplified in the prevention and control of dental caries, where “late stage” surgical intervention is the traditionally accepted management approach from the outset, and where early, pre-cavitated lesions are detected, they are often flagged for observation, “watch & wait”. Yet there are gaps in the philosophies and management modalities for oral diseases.

Active surveillance and early detection of periodontal disease are cornerstones of integrated person-focused oral health management. In periodontal disease management, the approach of “watch and wait” for early-stage manifestation of disease, and late-stage disease treatment are the antithesis of best practice principles. Applying the definitions of oral health in a comprehensive and consistent manner through a model for sports dental medicine would strengthen a planet-centric, health promoting and prevention focussed model of oral health services and care and encourage a holistic, salutogenesis approach health as well as to the use of natural resources and waste management to ensure no breach of planetary health boundaries.

In many cases the reactive model of care is dependent on the athlete visiting a dental practice, which impacts training and competition schedules. Each dental restoration, crown, and root canal treatment leaves ecological footprints from material production to energy consumption to waste generation, as well as recovery footprints on the athlete's performance. Dental practices contribute significantly to carbon emissions primarily through materials use and energy-intensive procedures, where the health sector is responsible for 4%–5% of the emissions of greenhouse gases worldwide (7). The reactive model of care benefits neither the athlete's health and performance, nor safeguards the Earth's natural resources.

Sustainable oral health recognises that oral health is intrinsic and inseparable from general health and well-being, and that oral health care is an integral part of health and development from the very first moments of life and throughout the life course (8). Sustainable oral health approaches enable a person to maintain their oral health in the face of adversity, trauma, tragedy, threats, or significant sources of stress.

The German Association for Sports Dentistry (Deutsche Gesellschaft für Sportzahnmedizin, DGSZM) is adopting a salutogenesis approach to health and oral health, one which emphasises factors that bolster health and well-being in collaboration with other professionals (9). This approach will help drive a fundamental shift in conceptualisation, service delivery and provision of oral healthcare for elite athletes. At the same time sports dentistry organisations are partnering with other branches of sports medicine to address the impact of climate change on health and athletic performance, and sports training programmes.

### 2.2 Sports dental medicine and planetary health

**Figure 1:**
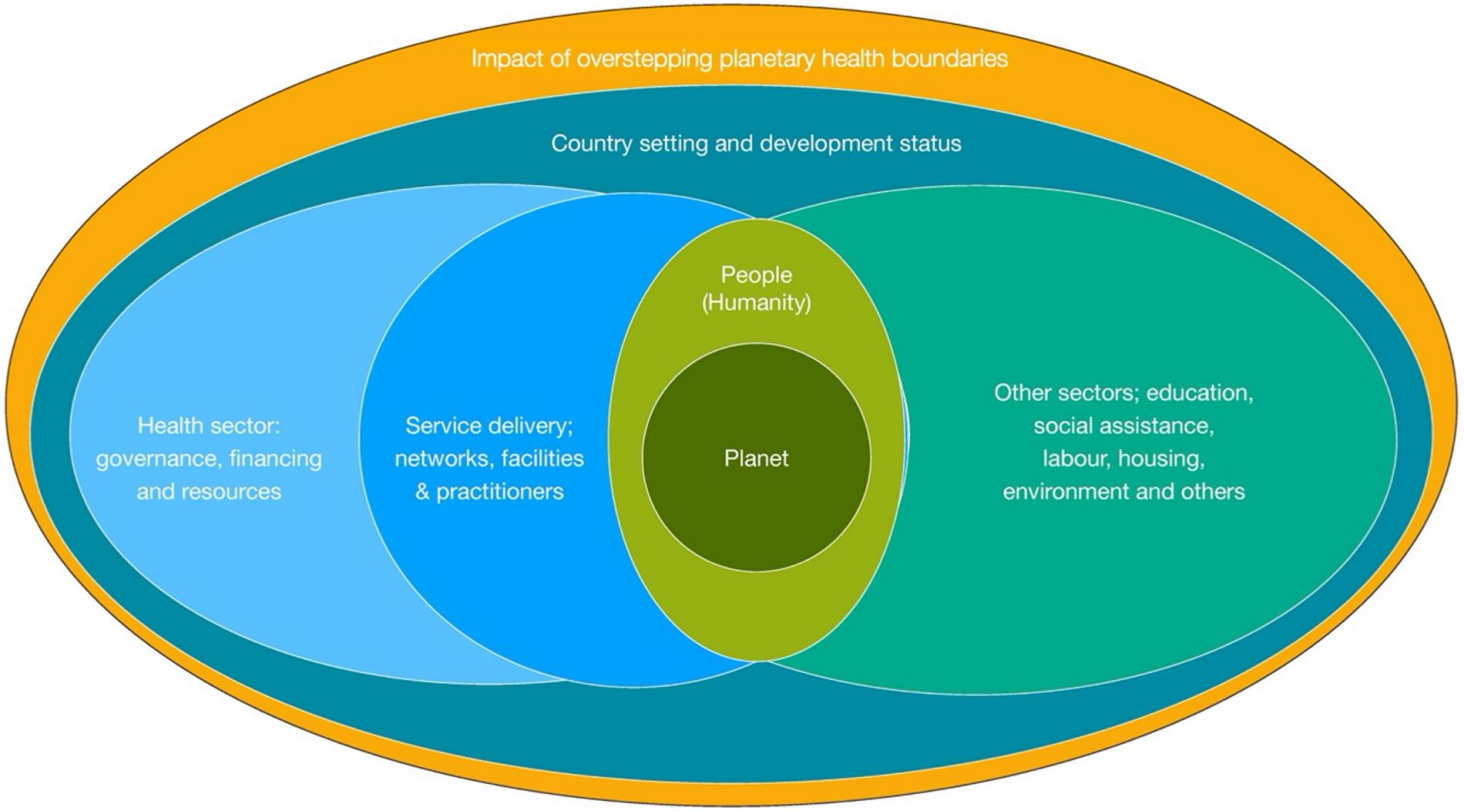
A model for oral and planetary health adapted from WHO framework on integrated people-centred health services.

The concept of planetary health is based on the understanding that a healthy environment -the conditions in which people are born, grow, work, live, and age is needed to support healthy communities. A model for oral and planetary health places more explicit focus on understanding the state of the Earth's systems, changing environment in relation to planetary health boundaries and their impact on human well-being (Figure 1) (10).

Sports dental medicine aligns well with the key tenets of sustainable dentistry. Sustainable dentistry can be described as practicing dentistry in ways that are environmentally friendly, socially responsible and accountable, and economically viable within planetary health boundaries and which safeguards the integrity of Earth systems. Sports dental medicine can contribute to advancing sustainable dentistry in general dental practice by providing an entry point for the concept of sustainable oral health which enables people to maintain their oral health in daily life and in the face of adversity throughout their lifespan, as well as promoting interprofessional and transdisciplinary partnerships for health and well-being. Furthermore, it may promote sports and exercise performance optimisation though functional analysis and therapy including identifying potential causes of snoring and sleep apnoea, as well as personalised dental splints (11).

Collaborative active surveillance, early detection, minimum intervention and holistic assessment, interprofessional and transdisciplinary collaboration that are supported by AI and digital technologies, are organisational principles that underpin sustainable oral health. Adopting these principles in general dental practice could substantially reduce dentistry's ecological footprint and improve oral health outcomes. Sports dental medicine, with its emphasis on health promoting and preventive focussed oral healthcare, real-time diagnostics, and collaboration provides an opportunity to pioneer and realise this transformation.

## 3 Artificial intelligence and biomimetic technologies in sports dental medicine

### 3.1 Artificial intelligence (AI)

Artificial Intelligence (AI) has the potential to power the next generation of oral health services and care and contribute to a more sustainable model of care (12). An AI-driven platform can facilitate enhanced disease detection but also link oral health data with musculoskeletal, respiratory, and metabolic parameters. Such an approach will enable sports dentists to contribute meaningfully to the optimisation of essential functions such as breathing efficiency, bite-force alignment, neuromuscular coordination, and recovery dynamics (13).

To operationalise the concept of sustainable oral health in sports medicine the Deutsche Gesellschaft für Sportzahnmedizin (DGSZM), Österreichische Gesellschaft für Sport- Zahnmedizin und Medizin (ÖGSZM) as well as Swiss Biological Medicine Centre (SBMC) and industry collaborators are co-developing an innovative AI-driven system that integrates multiple diagnostic and monitoring tools including multi-wavelength intraoral scans, intraoral OCT scanners, Cone Beam Computed Tomography (CBCT) imaging, salivary biomarkers (e.g., metalloproteinases) and mRNA analysis of oral microbiomes.

It aims to operationalise new ways of thinking and working to enable sports dentists to evolve from diagnosticians providing reactive care to highly specialised oral health coaches playing a key role in sports medicine aimed at sustaining peak athletic performance, delivering long-term health resilience and ensuring care remains with the planetary health boundaries. The adoption of AI can connect planetary boundaries with oral and planetary health. Although AII can foster more equitable and sustainable oral health it is important to consider both the positive impact and adverse effects of AI on oral and planetary health. Table 1 demonstrates how sustainable AI-enhanced oral health approach offers benefits for elite athletes and can contribute to the broader global oral health agenda.

**Table 1:** Alignment of sustainable AI-enhanced oral health approaches with sports dental medicine and the WHO global oral health action plan.

Sustainable AI-enhanced oral health approaches	Sports dental medicine applications	WHO global oral health action plan (strategic objective and action points)
Reduced invasive treatments and enhanced resource efficiency	Early detection enables non-invasive, as well as minimally invasive and environmentally friendly options through Minimum Intervention Oral Care (MIOC) delivery	Strategic objective 2, action point 37 Strategic objective 4, action point 67
Improved restoration longevity	Minimally invasive dentistry, repair vs. replacement, advanced diagnostics and preventive maintenance protocols can help reduce the frequency of re-interventions, clinical appointments and extend the functional lifespan of the tooth-restoration complex.	Strategic objective 4, action points 62, 67
Strengthen oral health information systems by unifying multiple data sources	Reduces duplicate examinations and their associated resource demands. Digital workflows, particularly intraoral scanning, eliminate the need for physical impressions, thereby reducing material waste, and allows a digital twin to be made available for diagnosis and long term active surveillance.	Strategic objective 5, action points 79, 80, 86
AI-enhanced strategic care planning, mid-stream and downstream promotion and prevention measures	Account for the athlete's full performance context: training load, recovery phases, breathing function, and musculoskeletal dynamics to guide preventive and therapeutic strategies.	Strategic objective 2, action points 29, 32 Strategic objective 5, action points 81, 82
Interprofessional and transdisciplinary communication and collaboration	Improved communication across health disciplines and can help remove traditional disciplinary boundaries in sports medicine to promote a collaborate approach to addressing the social and commercial determinants of health.	Strategic objective 1, action point 5 Strategic objective 2, action points 29, 30, 39 Strategic objective 4 action point 47

### 3.2 MOIC and biomimetic technologies

Minimum Intervention Oral Care (MIOC) is a holistic individualised framework that is cost-effective, prevention-based, susceptibility-related and oral healthcare team-delivered, encompassing four clinical domains (14, 15). These are i. early disease detection, diagnosis, susceptibility assessments, all leading to phased personalised care pathways, ii. prevention of lesions and control of disease (non-invasive, micro-invasive primary and secondary prevention approaches), iii. minimally invasive operative interventions (tertiary prevention) and iv. personalised recall and longitudinal active surveillance (16).

MIOC delivery can help guide the continuous development of Universal Screening Protocol for Dental Examinations in Sports (USPDES) to integrate oral health more fully into sport dental medicine surveillance and assessments (17).

Learning from nature has always played a pivotal role in propelling human scientific and technological advancements offering significant potential to revolutionise oral healthcare through innovative and nature-inspired solutions.

The clinical use of “biomimetic” regenerative materials such as self-assembling peptides is currently emerging in dentistry and shows promising results in terms of efficacy, effectiveness, ease of use and quality of life of patients (18). Non-invasive, technological innovations including digital infrared transillumination and intraoral OCT scanners can serve to specifically identify tooth surfaces at risk for developing caries, to aid carious lesion detection at the earliest stages of lesion development and to survey the progression or regression of a noninvasively treated caries lesion.

## 4 Discussion

Sports dental medicine offers a unique entry point to bridge oral health, sustainability, and planetary health. Collaborative active surveillance, early detection, primary, secondary, or tertiary prevention through non-, micro- and minimally invasive interventions, holistic assessment supported by AI and digital technologies are guiding and organisation principles that underpin sustainable oral health. Realising this potential will require stronger interprofessional collaboration, investment in AI and biomimetic technologies, and alignment with the WHO Global Oral Health Action Plan. If adopted at scale, sports dental medicine could catalyse a paradigm shift, shaping a future where oral health is health promoting and preventive focussed, sustainable, and safeguards the integrity of the Earth's systems.

## Data Availability

The original contributions presented in the study are included in the article/Supplementary Material, further inquiries can be directed to the corresponding author.
